# Machine learning-based predictive analysis of energy efficiency factors necessary for the HIFU treatment of adenomyosis

**DOI:** 10.3389/fphys.2025.1602866

**Published:** 2025-08-15

**Authors:** Ziyan Liu, Ziyi Liu, Yuan Wang, Xiyao Wan, Xiaohua Huang

**Affiliations:** Department of Radiology, Affiliated Hospital of North Sichuan Medical College, Nanchong, China

**Keywords:** adenomyosis, magnetic resonance imaging, high-intensity focused ultrasound, energy efficiency factor, prediction

## Abstract

**Purpose:**

This study aimed to develop a joint model combining T2-weighted imaging (T2WI) suppressed fat radiomics, and clinical parameters to predict the energy efficiency factor (EEF) required for high-intensity focused ultrasound (HIFU) ablation in patients with adenomyosis.

**Materials and methods:**

This retrospective study included 169 adenomyosis patients who underwent HIFU ablation between September 2021 and May 2024. EEF values were calculated based on T2WI fat suppression (T2WI-FS) sequences, and radiomics features were extracted. Predictive features were selected using minimum redundancy maximum relevance (MRMR) and least absolute shrinkage and selection operator (LASSO) methods, and two joint—based on decision tree and random forest algorithms—models were developed for EEF prediction.

**Results:**

The decision tree model achieved a mean absolute error (MAE) of 8.095 on the test set, while the random forest model exhibited an MAE of 8.231. The Wilcoxon rank-sum test for the test set revealed that the discrepancy in predictive performance between the two models was statistically significant (*p* < 0.05). The correlation coefficients were 0.768 and 0.777, and the *R* ^2^ coefficients of the two models in the test set were 0.559 and 0.549, respectively.

**Conclusion:**

The joint model integrating T2WI radiomics and clinical data effectively predicted EEF values for HIFU ablation in adenomyosis. This approach provides a foundation for optimizing HIFU dosing strategies and enhancing treatment safety and efficacy.

## 1 Introduction

Adenomyosis is a relatively common uterine disorder characterized by the abnormal invasion of endometrial epithelial cells and stromal fibroblasts into the myometrium (Zhai et al., 2020). Affected patients experience excessive uterine bleeding, pelvic pain, anemia, and infertility (Bulun et al., 2021), and the management of this condition often entails the use of surgical procedures, various drugs, uterine artery embolization, and high-intensity focused ultrasound (HIFU) ablation (Chinese experts' consensus on diagnosis and treatment of adenomyosis, 2020; Dason et al., 2023). However, pharmacological therapies are limited by incomplete efficacy and potential side effects (Shui et al., 2015). Surgical treatments may lead to endocrine disturbances and ovarian dysfunction, and hysterectomy is unsuitable for women desiring fertility (Li et al., 2021; Du et al., 2021). Furthermore, uterine artery embolization carries the risk of post-embolization syndrome (de Bruijn et al., 2016).

In patients, HIFU ablation offers particular utility as a fertility-preserving treatment option that is noninvasive and preserves fertility (Shui et al., 2015; Jeng et al., 2020; Siedek et al., 2019; Capezzuoli et al., 2024). HIFU treatment, however, is not an option for all patients, and differences in the amount of ultrasound required or the degree of therapeutic efficacy are evident among different adenomyosis pathological types (Gong et al., 2016; Yu et al., 2023). The energy necessary to ablate a unit volume (mm^3^) during HIFU procedures is referred to as the energy efficiency factor (EEF) (Gong et al., 2016; Li et al., 2006). Lower EEF values coincide with reduced energy requirements, more efficient ablation, and a shorter procedural duration, while higher EEF values necessitate more energy to achieve the same degree of ablation, resulting in longer procedures and increased risk of serious adverse events for patients (Wei, 2021). Precise control HIFU dosing strategies is thus vital for optimizing therapeutic efficacy and ensuring patient safety. When doses are not sufficient, a second treatment round may be necessary, whereas excessively high doses can cause nerve damage or cutaneous burns (Zhou, 2021).

MRI is often used for the assessment of adenomyosis as it provides high levels of soft-tissue resolution and is amenable to multiparametric multi-sequence imaging (O'Shea et al., 2020). Radiomics is a high-throughput, quantitative approach to processing large volumes of imaging data, enabling the detection of the heterogeneity of tissues of interest through feature extraction to support clinical decision-making (Lambin et al., 2017). So far, there have been relatively few studies exploring the use of T2-weighted imaging (T2WI) fat suppression (T2WI-FS) sequence-based radiomics features as a means of guiding HIFU dose delivery in adenomyosis patients. In this study, radiomics and clinical feature-based machine learning models were developed to predict EEF values in order to provide an imaging-based means of guiding dose delivery for the HIFU-based management of adenomyosis.

## 2 Materials and methods

### 2.1 Patient selection

The hospital Medical Ethics Committee provided retrospective approval for this study, with the requirement for informed consent having been waived. For this study, data from patients who underwent HIFU treatment for uterine adenomyosis between September 2021 and May 2024 in our hospital were collected retrospectively. Patient selection was based on predefined inclusion and exclusion criteria.

Inclusion criteria were as follows: (1) adenomyosis diagnosed based on clinical and imaging results; (2) the absence of any relevant history of surgery or other interventions; (3) >18 years of age; (4) MRI performed within 3 days of preoperative and postoperative examinations; (5) complete clinical data.

Exclusion criteria were as follows: (1) the presence of uterine fibroids; (2) any active pelvic inflammation, or suspected/confirmed pelvic malignancies; (3) missing or poor-quality images; (4) women who were breastfeeding or pregnant; (5) comorbid heart, liver, kidney, and other organ failure.

Based on these criteria, 169 patients were retained for further analysis.

### 2.2 MRI parameters

A 3.0T superconducting magnetic resonance imaging machine (UIH uMR790) with a 12-channel body-phased array coil was used for all MRI scanning. Each participant was positioned supine with the head facing forward, and a sandbag was placed on the abdomen to minimize respiratory artifacts. The T2-weighted fast spin-echo sequence with fat suppression (T2WI-FS) was used for adenomyosis lesion delineation and subsequent radiomics feature extraction. The key imaging parameters for the T2WI-FS sequence included repetition time (TR), echo time (TE), layer thickness, matrix, and field of view (FOV). Specifically, the T2WI-FS sequence used for this study had the following settings: TR = 2867 ms, TE = 93 ms, layer thickness = 4 mm, layer spacing = 2 mm, FOV = 240 mm × 240 mm, and matrix = 256 × 100. Contrast was injected at a rate of 2 mL/s, and arterial images corresponding to the early, intermediate, and late phases were collected at 15, 30, and 45 s, respectively, following contrast injection. The injection dose was 0.1 mmol/kg.

### 2.3 Preoperative data analysis

Preoperative MRI data for the enrolled patients were analyzed by two clinicians with over 5 years of imaging experience, with discussion and consensus in cases of disagreement. Analyzed clinical data included distance from the abdominal wall, distance from the leading edge of the lesion to the skin, uterus position (anterior/posterior), lesion volume, body mass index (BMI), age, leukocytes, erythrocytes, hemoglobin, platelets, treatment duration, and delivered energy. The EEF was defined as the amount of ultrasound energy required per unit volume of ablation and calculated as follows: EEF = treatment dose/volume of the non-perfused area (j/mm^3^) (Du et al., 2021).

### 2.4 Image segmentation and radiomics feature extraction

Two radiologists with extensive diagnostic experience used 3D Slicer software to outline lesions in a layer-by-layer manner in the SPAIR sequence using preoperative T2WI-FS MRI results. In cases of adenomyosis involving the entire uterine wall, the edge of the region of interest (ROI) was maintained at an appropriate distance from the endometrium and perimetrium layer. “PyRadiomics” software, integrated into the 3D Slicer platform, was used to extract 1,223 features from T2WI-FS sequence ROIs, including various texture, shape, and first-order features. One-third of all objects were randomly extracted from T2WI-FS sequences, and the corresponding ROIs were re-contoured, with the interclass correlation coefficient (ICC) parameter used to examine consistency among investigators. These features with an ICC ≥0.75 were retained.

### 2.5 Feature selection and model development

The uAI Research Portal (v. 730) was used to analyze radiomics and clinical features separately and develop a predictive model. Initially, patients in the study population were randomized at a 7:3 ratio into the training (n = 118) and testing (n = 51) cohorts. Radiomics feature data analyzed via ICC were Z-score-normalized, and those features that were highly correlated with the EEF but not redundant were retained via minimum redundancy maximum relevance (MRMR), while the least absolute shrinkage and selection operator (LASSO) method was used to screen the retained features, thereby improving the model’s generalizability. Subsequently, L2 norm regularization was applied to the screened features during processing, and the final radiomics and combined regression models were developed using the decision tree and random forest methods. Model performance was assessed based on the *R* ^2^ coefficient, correlation coefficient, and mean absolute error (MAE) values.

### 2.6 Statistical analyses

Data were analyzed using R (version 4.3.2) and SPSS 27.0. Normally distributed and skewed continuous data were reported as *x ± s* and M (Q25, Q75), respectively, with count data instead being reported as the number of cases or the corresponding ratio. MAE values were compared among methods using the Wilcoxon rank-sum test.

## 3 Results

### 3.1 Patient data

In total, this study included 169 patients with a median age of 43 years (range: 21–56 years). Other baseline clinical data for these patients are presented in Table 1.

**TABLE 1:** Clinical baseline characteristics.

Variable	Value
EEF (J/mm^3^)	5.47 (2.69, 10.55)
BMI (kg/m^2^)	23.43 ± 2.66
Uterine position (anteverted/retroverted)	131/38
Distance of the abdominal wall (mm)	27.89 ± 7.20
Distance from the edge of the lesion to the skin (mm)	60.89 ± 22.12
Lesion volume (mm^3^)	68,929.76 (37,278.81, 114,587.85)
Leukocytes (10^9^/L)	5.94 ± 1.56
Erythrocytes (10^12^/L)	4.30 ± 0.53
Platelets (10^9^/L)	254.00 (204.50, 320.50)
Hemoglobin (g/L)	111.00 (95.00, 128.00)
Treatment duration(s)	608.00 (408.00, 835.00)
Delivered energy(J)	243,200.00 (162,600.00, 334,000.00)

### 3.2 Feature selection

Based on ICC analyses, 1,040 T2WI-FS sequence features were retained for analysis (ICC > 0.75). Using the MRMR method for preliminary feature selection (Table 2), followed by the LASSO method for further refinement, the most important radiomics and clinical features for predicting HIFU ablation doses were selected, resulting in the retention of 12 radiomics features and 3 clinical features, namely, lesion edge-to-skin distance, lesion volume, and red blood cell count (Table 3).

**TABLE 2:** Clinical features after selection through MRMR.

Feature	Score
Lesion volume	0.204
Erythrocyte	0.028
Distance from the edge of the lesion to the skin	0.019
BMI	0.019
Years	0.014

**TABLE 3:** Radiomics and clinical features selected through LASSO.

Type	Feature	Coefficient
—	Distance from the edge of the lesion to the skin	2.82
—	Lesion volume	−4.95
—	Erythrocyte	−0.06
Original shape	Sphericity	−3.80
log-sigma-20mm-3D-glcm	Idn	−1.20
log-sigma-20mm-3D-ngtdm	Coarseness	4.59
wavelet-LLH-gldm	DependenceVariance	2.34
wavelet-LHL-glcm	MCC	6.27
wavelet-HLH-glrlm	RunLengthNonUniformityNormalized	1.45
wavelet-HHL-firstorder	Median	0.40
wavelet-HHL-ngtdm	Contrast	1.94
wavelet-HHH-firstorder	Median	−1.77
wavelet-HHH-gldm	SmallDependenceEmphasis	1.76
wavelet-HHH-glrlm	RunVariance	−1.44
wavelet-HHH-glszm	GrayLevelNonUniformityNormalized	0.72

Original-shape sphericity: this is used to measure the similarity of the tumor shape to a perfect sphere.

log-sigma-20mm-3D-glcm_Idn: this is derived from the gray-level co-occurrence matrix (GLCM) calculated on 3D images filtered using a Laplacian of Gaussian (LoG) filter with a sigma of 20 mm. The inverse difference normalized (Idn) quantifies local homogeneity.

log-sigma-20mm-3D-ngtdm_Coarseness: this is computed from the neighborhood gray-tone difference matrix (NGTDM) on LoG-filtered images at the same scale; this feature measures texture coarseness, indicating the fineness or roughness of tissue structure.

wavelet-LLH-gldm_DependenceVariance: this is extracted from images processed with wavelet decomposition at low–low–high (LLH) frequencies; this gray-level dependence matrix (GLDM) feature measures variance in dependence counts.

wavelet-LHL-glcm_MCC: this is the maximal correlation coefficient (MCC) from GLCM of wavelet-filtered images at low–high–low (LHL) frequencies, assessing texture correlation complexity and tumor heterogeneity.

wavelet-HLH-glrlm_RunLengthNonUniformityNormalized: this is calculated from the gray-level run length matrix (GLRLM) of high–low–high (HLH) wavelet sub-band images; this normalized run length non-uniformity reflects irregularity and complexity of texture patterns.

wavelet-HHL-firstorder_Median: this is the median gray-level intensity from first-order statistics of high–high–low (HHL) wavelet images, associated with tissue density.

wavelet-HHL-ngtdm_Contrast: this is the contrast feature derived from the NGTDM on HHL wavelet images, indicating gray-level differences.

wavelet-HHH-firstorder_Median: this is the median intensity of the high–high–high (HHH) wavelet sub-band images, describing the central tendency of gray levels.

wavelet-HHH-gldm_SmallDependenceEmphasis: this is a GLDM measure emphasizing small-dependence regions, capturing fine structural details.

wavelet-HHH-glrlm_RunVariance: this is the variance of run lengths from the GLRLM on HHH images, reflecting texture heterogeneity;

wavelet-HHH-glszm_GrayLevelNonUniformityNormalized: this is the normalized gray-level non-uniformity from the gray-level size zone matrix (GLSZM), quantifying the variability of gray-level values in texture.

### 3.3 Model results

The combined models based on 12 imaging features and 3 clinical features were developed using decision tree and random forest methods. These two models demonstrated MAE values of 8.095 and 8.231, respectively, in the testing cohort; the Wilcoxon rank-sum test revealed a statistically significant difference in predictive efficacy between the models in both the training and testing cohorts (*p* < 0.05). The Pearson correlation coefficients for the two models in the testing cohort were 0.768 and 0.777, respectively. Both models showed a strong correlation between the predicted and actual EEF values, indicating that the developed EEF dose prediction models have practical value (Table 4).

**TABLE 4:** Predictive performance results of the two models.

Model	MAE	*R* ^2^ coefficient	Correlation coefficient
Decision tree			
Training set	9.725	0.508	0.713
Test set	8.095	0.559	0.768
Random forest			
Training set	9.934	0.536	0.779
Test set	8.231	0.549	0.777

## 4 Discussion

As a noninvasive treatment modality, HIFU has emerged as an important strategy for adenomyosis treatment as it can help preserve patient fertility (Jeng et al., 2020; Lee, 2021; Wei et al., 2023). The energy dose used for HIFU ablation is closely correlated with procedural efficacy, and the EEF, which measures the energy necessary to ablate a unit volume of the target lesion, is a key index associated with HIFU therapeutic dosing (Yu et al., 2023; Xu et al., 2023). Precise control of patient doses is essential to ensure that treatments are safe and efficacious. If the dose is insufficient, the ablation effect may be inadequate, potentially requiring an additional round of treatment, whereas excessive dosing may result in adverse outcomes such as cutaneous burns or nerve damage (Wei, 2021). To ensure that the appropriate amount of energy is deposited in the target area, EEF thus needs to be precisely predicted and controlled, allowing for the optimization of therapeutic efficacy while preventing complications. In this study, a combined model based on 12 radiomics features derived from T2WI-FS images and 3 clinical features was developed using the decision tree and random forest methods to facilitate EEF prediction in the context of the HIFU ablation-based treatment of adenomyosis. Both of these models performed well in the training and testing cohorts, with the combined model exhibiting preferable predictive ability such that it may aid clinicians in their efforts to develop a reasonable approach to treating adenomyosis patients.

In some reports, researchers have demonstrated the utility of clinical and imaging features as predictors of HIFU ablation dose in patients with uterine diseases. Gong et al. (2016) determined that the ventral distance from the skin of uterine adenomyosis foci, T1WI enhancement type, and foci volume were linearly associated with EEF such that they may be leveraged for a predictive index for the HIFU-based treatment of uterine adenomyosis. Peng et al. (2015) explored the factors that impact ultrasound dose and developed a HIFU dosing model for uterine fibroid treatment, ultimately determining that the ventral distance from the skin to the fibroids and T2WI signal intensity could serve as effective dosimetric predictors in this context. Conventional MRI features derived from visual image inspection, however, are susceptible to inter-observer variability due to differences in experience, compromising the accuracy and stability of these results. Radiomics strategies, in contrast, can enable the high-throughput extraction of quantitative imaging data and the detection of minute differences among images not visible to the naked eye that can be used for further analyses (Lambin et al., 2017; Gillies et al., 2016). Accordingly, a combined model was developed in this study based on MR radiomics features and clinical features to guide the prediction of EEF values for the HIFU-based treatment of adenomyosis. The results are expected to offer guidance for therapeutic dosing efforts. Relative to the T1WI sequences, T2WI-FS sequences do not necessitate the use of contrast, which can damage the kidneys and liver, and signal changes for this sequence are related to the pathological features of uterine adenomyosis (Du et al., 2021; Yu et al., 2023). The T2WI-FS sequence is also closely associated with the pathophysiological changes observed in adenomyotic foci, prompting the selection of this sequence for radiomics feature extraction in the present study.

In this study, the distance from the leading lesion edge to the skin and the lesion volume were identified as predictors of the EEF for HIFU ablation, in line with prior studies (Gong et al., 2016). The greater the distance between the lesion’s leading edge and the skin, the greater the degree to which ultrasound waves will be subjected to reflection, absorption, and scattering during propagation, resulting in attenuation that can adversely affect ablation therapy (Gong et al., 2016; Baker et al., 2001). Deeper focal points have a greater effect on ultrasound during propagation, particularly affecting the tissues in front of the focal point, resulting in the attenuation of the ultrasound energy. Deeper foci require a larger amount of ultrasound energy to achieve the same volume of necrosis relative to superficial lesions with the same ablation rate. This is because tissue interfaces in the acoustic channel may result in greater energy attenuation owing to the absorption, reflection, and scattering of ultrasound waves with increasing volume in front of the lesion (Peng et al., 2015; Liu et al., 2018). As adenomyotic lesions become larger, a lower dose is necessary to ablate a given target tissue volume. This is attributable to the larger lesions occupying a larger amount of the acoustic pathway, minimizing sciatic nerve pain, and decreasing the interval between acoustic treatments such that greater amounts of acoustic energy can be delivered in a given amount of time, thereby preventing the treatment area from cooling. As necrotic tissues have a dynamic impact on the acoustic conditions within the target lesion, this may also play a role in ultrasound energy deposition (Liu et al., 2018; Chen et al., 1997). Additionally, this study found a significant negative correlation between erythrocyte count and the EEF required for HIFU treatment of adenomyosis. Specifically, higher erythrocyte counts were associated with a lower required EEF. We hypothesize a possible mechanism for this phenomenon: an increase in the erythrocyte content alters the acoustic properties of tissue, particularly the acoustic impedance and the propagation path of ultrasound waves in blood-rich tissues. As erythrocyte density increases, the absorption and focusing efficiency of ultrasound energy within the tissue are enhanced, thereby improving energy utilization and reducing the total energy required to achieve therapeutic effects. Furthermore, a high erythrocyte concentration may alter the way heat is deposited in the local microenvironment, making it easier for the treatment area to reach the desired temperature threshold, thus improving the efficiency of the treatment outcome.

The advantage of this study is the development of a clinically applicable predictive model that may facilitate the optimization of ultrasound energy dosing by enabling preoperative adjustment of treatment parameters, thereby improving procedural efficiency and reducing the likelihood of prolonged treatment durations or the need for repeat ablations due to under-dosing. By providing a more precise estimate of the energy required to ablate a specific lesion volume, the model also helps prevent excessive energy delivery, which, in turn, reduces the risk of complications and enhances overall treatment safety. Importantly, by striking an appropriate balance between sufficient and excessive dosing, this approach not only safeguards patient wellbeing but also promotes greater treatment compliance, satisfaction, and long-term clinical benefit.

This study has several limitations. The retrospective design may introduce selection bias. Furthermore, the lack of external validation and the relatively small sample size constrain the generalizability of the findings. Although the model achieved moderate predictive performance (*R* ^2^ = 0.50–0.55), providing a clinically meaningful benchmark, significant room for improvement remains in HIFU’s EEF modeling. Additionally, variations in MRI equipment and acquisition protocols across institutions pose a major challenge to the reproducibility of radiomics features, potentially compromising the model’s clinical applicability. To address these limitations, future research will focus on expanding the sample size and conducting multicenter studies to evaluate the model’s generalizability and robustness across diverse populations and clinical settings. We will also explore advanced ensemble learning methods, such as XGBoost and LightGBM, to further enhance predictive accuracy. Moreover, efforts will be undertaken to standardize MRI protocols and calibrate equipment consistently across all participating centers. These initiatives aim to enhance the model’s reliability and clinical utility, enabling its use as a tool to guide clinical decision-making in HIFU-based treatments for adenomyosis.

## 5 Conclusion

In summary, the T2WI-FS radiomics and clinical feature-based model developed in this study was able to reliably predict the EEF value necessary for the HIFU ablation of uterine adenomyosis. This tool can thus effectively guide the clinical assessment of the difficulty of HIFU-based uterine adenomyosis treatment, providing a reference for HIFU dose selection while improving the safety and efficacy of this interventional approach.

## Data Availability

The original contributions presented in the study are included in the article/Supplementary Materials, further inquiries can be directed to the corresponding author/s.
